# The Legal Control of Medical Practice by a State Examination

**Published:** 1885-04

**Authors:** 


### The Legal Control of Medical Practice by a State Examination.

There is, on all sides, a growing sentiment in favor of divorcing the teaching and licensing power, in reference to fixing the legal status of the physician; or, in other words, the public mind is becoming more and more convinced that the diplomas granted by the medical colleges should not, likewise, confer the legal right to practice medicine.

At first glance it would seem almost strange that the State has not assumed control of this matter before, involving, as it does, questions relating to the life, health and longevity of its citizens. It already prescribes special examinations for persons entering the public service in a medical capacity, showing a jealous care in that respect in its administration of public trusts. Why should it longer neglect the private citizen by refusing to protect him from incompetent, ignorant, sordid and criminally indifferent medical service? The prosperity of the State means the prosperity of the individual, his protection in life, liberty and the peaceful pursuit of his avocation. He should be instructed, through an intelligent medical source, in the laws of hygiene, sanitation, and the general methods and habits of life, which apply to the prevention of disease. It is safe to say that none of the great powers of the world pay so little attention to the protection of health and life as does our own loved and cherished republic. In other countries the university degree does not confer the right to practice, but its holder must also pass a government examination before he is admitted to the exercise of duties, which involve the guardianship of the health of the citizens of the empire.

In most European countries, though the universities are under the supervision of the State; their degrees are not considered as sufficient evidence that the holders thereof are qualified to practice medicine, but they must invariably submit to the further examination by a Board of Examiners appointed by the State, who are in every way separate and distinct from the teaching power.

Here, in this country, the medical schools are dependent for prosperity upon the amount paid in fees by the pupils who attend, and just so long as examination by faculties, who receive these fees, are accepted as the sole evidence of qualification to practice, their diplomas will possess little value as an index of true professional skill or learning. What is required is a uniform standard for the establishment of the right to practice medicine, and it seems impossible to obtain this, except by the intervention of the State through an Examining Board of its own creation. As it now is, each medical college fixes its own standard as to curriculum and requirements for its degree; with some this may be high, with others low, yet all be within the technical requirements of the law. There are a few medical schools, one notable exception being in our own city, which, appreciating the trend of public sentiment in favor of a more complete preparation for the medical calling, have advanced their standard in compliance with this demand. Let the State once create an Examining Board, and all will move up to the front line.

The great hospitals in New York, and other cities, long ago adopted a similar plan of examining candidates for appointments as resident physicians, the governing boards not being willing to accept the diplomas of the schools, as evidence of the possession of sufficient skill or learning on the part of the applicants to entrust to their care the unfortunate inmates of their wards. What better evidence could any one ask for the necessity of a State Licensing Board?

The chief difficulties in the way of the accomplishment of the desired end, seem at present to reside in the want of harmony between the different so-called sects or systems of medicine, on this subject. The average legislator appears to fear to touch the question on this account. But this whole matter should be considered quite secondary to the important object to be attained. Moreover, this obstacle can readily be surmounted by committing to the Board power to examine in anatomy, physiology, chemistry, pathology, surgery, obstetrics, materia medica, and hygiene only, leaving therapeutics and all theories of medical practice entirely out of the field. To this, not even the most ardent advocate of special systems or codes could object, for the law would remain silent on these vexed questions, permitting the candidate to receive his education in any school, and come before a Board which had nothing to do with teaching him, and could, therefore, have no bias in reference to passing him.

